# Proteomic and genomic analysis reveals novel *Campylobacter jejuni* outer membrane proteins and potential heterogeneity

**DOI:** 10.1016/j.euprot.2014.06.003

**Published:** 2014-06-26

**Authors:** Eleanor Watson, Aileen Sherry, Neil F. Inglis, Alex Lainson, Dushyanth Jyothi, Raja Yaga, Erin Manson, Lisa Imrie, Paul Everest, David G.E. Smith

**Affiliations:** a Moredun Research Institute, Bush Loan, Penicuik, United Kingdom; b Institute of Infection, Immunity and Inflammation, College of Medical, Veterinary and Life Sciences, University of Glasgow, Glasgow, United Kingdom

**Keywords:** *Campylobacter jejuni*, Proteomics, Outer membrane, Genomics

## Abstract

Gram-negative bacterial outer membrane proteins play important roles in the interaction of bacteria with their environment including nutrient acquisition, adhesion and invasion, and antibiotic resistance. In this study we identified 47 proteins within the Sarkosyl-insoluble fraction of *Campylobacter jejuni* 81-176, using LC–ESI-MS/MS. Comparative analysis of outer membrane protein sequences was visualised to reveal protein distribution within a panel of *Campylobacter* spp., identifying several *C. jejuni*-specific proteins. Smith–Waterman analyses of *C. jejuni* homologues revealed high sequence conservation amongst a number of hypothetical proteins, sequence heterogeneity of other proteins and several proteins which are absent in a proportion of strains.

## Highlights

• Identification of 47 *Campylobacter jejuni* outer membrane proteins by LC–ESI-MS/MS.
• Bioinformatic analysis reveals *C. jejuni* specific proteins.
• Sequence heterogeneity within *C. jejuni* OM proteins is displayed graphically.

## Graphical abstract

**Figure:**
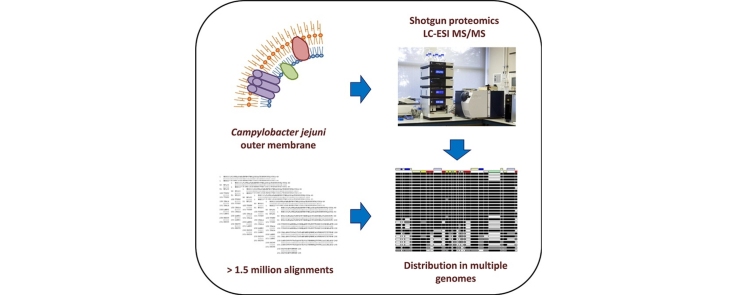

## 1 Introduction

*Campylobacter* spp. are the commonest cause of food-borne disease worldwide accounting for 2.4 million cases per year in the US (www.cdc.gov/foodnet) with *Campylobacter jejuni* and *C. coli* responsible for the majority of infections. In the developing world these organisms are the leading cause of gastrointestinal infection in children under 2 years of age [1]. Additionally, post-infection sequelae may arise including Guillain–Barré syndrome (GBS) and other debilitating neurological disorders 2, 3. Despite a significant worldwide effort, mechanisms of disease and immunity remain poorly understood. Gram-negative bacterial outer membrane (OM) proteins represent a group of factors which play important roles in the interaction of bacteria with their environment. These include porins and nutrient uptake systems, iron acquisition proteins, virulence factors, proteins involved in antibiotic resistance and other proteins required for survival within the hostile *in vivo* environment.

To date, functional roles have been ascribed to only a dozen or so *C. jejuni* OM proteins. A more comprehensive characterisation of this important group of proteins, even in prototypic strains, has yet to be reported. Several *C. jejuni* surface proteins have been observed to play roles in adhesion. Three of these, CadF and FlpA, and PEB1 have been shown to play a role in the colonisation of broiler chicks 4, 5. Additionally, JlpA, CapA and PorA appear to mediate binding to epithelial cells in culture 6, 7, 8. Another important role is the transportation of small molecules across the bacterial cell membrane. *C. jejuni* lacks the phosphofructokinase protein and cannot therefore metabolise glucose although recently some strains were shown to use fucose as a substrate for growth 9, 10. Instead, the bacterium utilises amino acids as a source of carbon and energy; the surface proteins PEB1, and CjaA are components of ABC transporters with roles in aspartate/glutamate, and cysteine uptake respectively 11, 12. OM efflux systems are also important: CmeC and CmeD are OM components of two functionally characterised multi-drug efflux pumps, which have roles in antimicrobial and bile resistance 13, 14. Omp50 and PorA have been characterised as porins and the former was recently shown to play a role in phosphotyrosine network regulation 15, 16, 17. *Campylobacter* spp. also possess a number of iron acquisition systems, components of which reside in the OM including the Fe^3+^ enterobactin binding proteins CfrA and CfrB 18, 19, 20 and the heme transporter ChuA, [21]. Flagella have a variety of proposed roles in *C. jejuni* virulence and protein components which anchor flagella to the membrane (FlgH) are located in the OM [22]. OM proteins may also elicit pathological host responses; the surface-exposed lipoprotein JlpA is believed to trigger signalling events, which lead to inflammation [23].

Interrogation of the *C. jejuni* genome has revealed that a number of protein complexes and chaperones, which are essential for membrane biogenesis in many well characterised Gram-negative bacteria, are either divergent or absent altogether within *C. jejuni* genomes [24]. The β-barrel assembly machinery is known as the Bam complex [25]. The closest homologue of the OM localised component, BamA (formerly known as YaeT), is divergent in *C. jejuni* and, to the best of our knowledge, homologues of BamBCDE, are not found at all within *Epsilonproteobacteria* genomes. LolB of the Lol (lipoprotein localisation) complex, which is responsible for sorting lipoproteins [26] is absent in *Epsilonproteobacteria*, although homologues of other proteins within this complex are present in *C. jejuni* genomes. *C. jejuni* also varies from the majority of other Gram-negative bacteria in that it lacks the O-antigen of LPS. Often referred to as LOS (lipooligosacharide), this is perhaps a reflection of the divergence of the Lpt (*LP*S *t*ransport) proteins which are required for insertion of LPS into the outer membrane. This suggests that alternative mechanisms of maintaining membrane integrity and biogenesis have yet to be revealed within *C. jejuni* and related bacteria. Existing evidence suggests that distinct mechanisms for OM biogenesis exist outside the *Gammaproteobacteria* group of bacteria 27, 28. The divergence in this cellular machinery for protein sorting also suggests that conventional *in silico* protein localisation tools may not be reliable for this particular group of bacteria and therefore localisation of these proteins by experimental methods is particularly pertinent.

Approximately one third of *C. jejuni* predicted open reading frames (ORFs) code for proteins of unknown function and it is unlikely that the current list of characterised OM proteins is complete. Therefore, a thorough analysis of expressed OM proteins is essential for identifying factors important to and possibly novel to *C. jejuni* pathogenesis. We performed proteomic analysis of the OM of *C. jejuni* subsp. *jejuni* 81-176, a widely employed strain which causes experimentally reproducible clinical disease [29]. A variety of methods have been used previously for membrane protein enrichment although the fractions of extracted proteins vary in composition. The most comprehensive proteome analysis of *C. jejuni* to date focussed on the entire membrane compartment, *i.e.* periplasm and both inner and outer membranes [30]. *Campylobacter* OM appears to be closely associated with the inner membrane and as a result is more challenging to purify [31]. Recently, Hobb et al. [32] reported that *N*-lauroylsarcosine (Sarkosyl) treatment of *C. jejuni* cells was the most successful method of isolating specifically OM proteins. Furthermore, Sarkosyl enrichment is often used to predict localisation of *Campylobacter* proteins 15, 33, 34.

The low solubility of OM proteins renders them incompatible with the majority of proteomic techniques hence gel-based proteomics offers a convenient method for their analysis as issues associated with hydrophobicity are circumvented by ionic detergent (sodium dodecyl sulfate) solubilisation and subsequent in-gel tryptic digestion 35, 36. In this study, a rapid shotgun proteomics-based approach was used to catalogue the protein complement of the *C. jejuni* OM fraction. This methodology, comprising SDS-PAGE, one-dimensional monolithic column liquid chromatography, electrospray ionisation (ESI) and fast MS/MS scanning, is colloquially termed “sawn-off shotgun proteomic analysis” (SOSPA). This approach enables the analysis of membrane-associated and other hydrophobic proteins whilst simultaneously combining rapidity with breadth of coverage. Bioinformatic approaches were deployed to survey the resulting SOSPA-generated data to identify homologous proteins amongst bacterial, *Epsilonproteobacteria* and, particularly, *Campylobacter* genomic sequences.

## 2 Materials and methods

### 2.1 Bacterial strains, media and culture conditions

*C. jejuni* 81-176 (pVir+) is a well characterised strain, isolated from contaminated milk [37]. Bacteria were grown at 37 °C in a variable atmosphere incubator (Don Whitley Scientific, Shipley, UK) in an atmosphere of 6% hydrogen, 5% carbon dioxide, 5% oxygen, and 84% nitrogen. Bacteria were cultured for 48 h on *Campylobacter* selective agar (Skirrow) plates (E&O Laboratories, Bonnybridge, UK) then resuspended in 20 ml high glucose Dulbecco's modified Eagle's medium (cat. 11960, Invitrogen, Paisley, UK), supplemented with 20 mM l-glutamic acid and 0.00125% iron ascorbate (Sigma, Dorset, UK) [38] at an Abs_600_ of 0.1. Cultures were incubated statically for 34 h; the timepoint was chosen to represent growth in late log phase.

### 2.2 OM protein enrichment

OM protein enrichment was carried out as described by Gauthier et al. [39]. Bacteria from broth cultures were pelleted at 8000 × *g* and resuspended in 500 μl of 50 mM Tris–HCl (pH 7), with 20% sucrose, 10 μM EDTA, 10 μg/ml lysozyme and protease inhibitors (Complete Mini EDTA Free, Roche Diagnostics Ltd, Burgess Hill, UK) for 10 min at room temperature. All subsequent steps were carried out on ice. Bacteria were centrifuged at 8000 × *g* for 10 min and the supernatant removed. The pellet was resuspended in 1 ml Tris buffer (10 mM Tris–HCl, pH 7) with protease inhibitors and sonicated on ice (amplitude 5.0, 6 × 15 s). Unbroken bacteria were removed by centrifugation at 16,000 × *g* for 2 min and the supernatant was centrifuged for 1 h at 50,000 × *g* to pellet bacterial membranes. Pelleted membranes were washed with Tris buffer, resuspended in Tris–Sarkosyl buffer (Tris buffer containing 0.5% (w/v) *N*-lauroylsarcosine), and centrifuged for 1 h at 50,000 × *g*. The OM pellet was washed in Tris–Sarkosyl buffer then resuspended in Tris–Sarkosyl buffer containing 0.1% SDS.

### 2.3 LC–ESI-MS/MS

Sarkosyl-insoluble proteins, prepared as described above, were resolved on a 4–12% Bis–Tris NuPAGE SDS-PAGE gradient gel (Invitrogen, Paisley, UK) in MES Buffer at 200 V (constant voltage) over 45 min. Proteins were visualised using Colloidal Coomassie Blue G250 (Sigma, Dorset, UK). Molecular size standards were included routinely on gels. LC–ESI-MS/MS was carried out essentially as described by Batycka et al. [40]. For each sample lane, a series of gel slices of equal size (2.5 mm), covering the entire lane, were excised from the SDS-PAGE gel before performing standard in-gel de-staining, reduction, alkylation and trypsinolysis procedures [41]. The samples were transferred to HPLC sample vials and stored at +4 °C until required for LC–ESI-MS/MS analysis. Liquid chromatography was performed using a Dionex Ultimate 3000 nano-HPLC system (Thermo Fisher Scientific, Hemel Hempstead, UK) comprising a WPS-3000 well-plate micro auto sampler, a FLM-3000 flow manager and column compartment, a UVD-3000 UV detector, an LPG-3600 dual-gradient micropump and an SRD-3600 solvent rack controlled by Chromeleon chromatography software (www.thermoscientific.com/dionex). A micro-pump flow rate of 246 μl/min^−1^ was used in combination with a cap-flow splitter cartridge, affording a 1/82 flow split and a final flow rate of 3 μl/min^−1^ through a 5 cm × 200 μm ID monolithic reversed phase column (Thermo Fisher Scientific, Hemel Hempstead, UK) maintained at 50 °C. Samples of 4 μl were applied to the column by direct injection. Peptides were eluted by the application of a 15 min linear gradient from 8% to 45% solvent B (80% acetonitrile, 0.1% (v/v) formic acid) and directed through a 3 nl UV detector flow cell. LC was interfaced directly with a 3-D high capacity ion trap mass spectrometer (Esquire HCTplus™, Bruker Daltonics, Bremen, Germany) *via* a low-volume (50 μl/min^−1^ maximum) stainless steel nebuliser (Agilent Technologies, Wokingham, UK; cat. no. G1946-20260) and ESI. Parameters for tandem MS analysis were set as previously described [40]. Technical controls included BSA standard and a blank gel slice.

### 2.4 Database mining

Deconvoluted MS/MS data were searched against an annotated cognate chromosomal and plasmid *C. jejuni* 81-176 protein database derived from genomic sequences available at the National Centre for Biotechnology Information (Genbank), http://www.ncbi.nlm.nih.gov (Table 1) and the NCBInr *C. jejuni* sub-database, using MASCOT software (Matrix Science, London, UK) [42]. Analysis was performed in accordance with published guidelines [43]. To this end, fixed and variable modifications selected were carbamidomethyl (C) and oxidation (M) respectively and mass tolerance values for MS and MS/MS were set at 1.5 Da and 0.5 Da respectively, permitting one missed cleavage. Positive protein identifications were based on recognition of a minimum of two peptides, each with an unbroken series of four or more “*b*” or “*y*” ions [40]. Deconvoluted MS/MS data in .mgf (Mascot Generic Format) were imported into ProteinScape™ proteomics data analysis software (Bruker Daltonics, Bremen, Germany) which compiles data from all gel slices utilising the MASCOT search algorithm (Matrix Science, London, UK). The protein content of individual gel slices was established using the “protein search” feature of ProteinScape™, whilst separate compilations of the proteins contained in all 25 gel slices of each of the three biological replicates were produced using the “protein extractor” feature of the software. Data was searched specifying Trypsin and Trypsin/P. Spectra used for protein identifications were re-searched against the entire NCBInr database to ensure accurate peptide assignments.

### 2.5 Amino acid sequence comparison tools

Protein sequences were compared by two methods. First, an in-house Java application which takes a single reference genome in protein multi-FastA and annotation information in .ptt formats (http://www.ncbi.nlm.nih.gov/sites/genome), plus several user-defined comparison genomes in protein multi-FastA format. This tool uses BLASTP [44] to define putative orthologs between reference and comparison genomes and displays a graphical alignment of these proteins showing alignment score, metadata and an amino acid alignment. The *Campylobacter* genomes examined are listed in Table 1. NCBI genome datasets comprised (i) *Epsilonproteobacteria* class, excluding the *Campylobacter* genus, and (ii) all bacterial genomes excluding the *Epsilonproteobacteria* class. Second, Smith–Waterman global alignments [45] were carried out between protein pairs. Smith–Waterman scores were normalised using the score for identical sequences as reference (100%) to take account of the disparity in protein length. Scores were displayed as a heat map.

### 2.6 Bioinformatic tools

The LipoP 1.0 Server (http://www.cbs.dtu.dk/services/LipoP/) was used to predict N-terminal signal peptides in lipoproteins [46]. The β-barrel outer membrane protein predictor (BOMP) server (http://services.cbu.uib.no/tools/bomp) and Phyre2 (**P**rotein **H**omology/analog**Y R**ecognition **E**ngine V 2.0) (http://www.sbg.bio.ic.ac.uk/phyre2/), were used to predict the presence of β-barrels within protein sequences 47, 48. Functional domains and motifs were identified in protein sequences using Interproscan server (www.ebi.ac.uk/interpro/) using all available methods [49].

## 3 Results and discussion

### 3.1 LC–ESI-MS/MS identification of *C. jejuni* strain 81-176 Sarkosyl-insoluble proteins

Strict confidence criteria were used when assigning peptide and protein identities [43]. To ensure reproducibility, only proteins represented by two peptides in at least two of three replicates are presented here (Table 2). Sequence coverage was determined using only top ranking peptides. Using the Trypsin/P setting of ProteinScape™, which allows detection of peptides generated by occasional trypsin digestion between lysine or arginine and proline [50], 8 additional peptides were identified, including one which defined l-lactate permease. All matches are found in Tables S1–S6 of the supplementary data.

Applying those criteria, 47 proteins were identified confidently within the Sarkosyl-insoluble material. These comprised several previously characterised OM proteins including the adhesins CadF and FlpA, the MapA antigen and a number of proteins with known roles in small molecule transport including the porins PorA and CJJ81176_1185 (Omp50), ABC transporter components PEB1, CjaA and CjaC and the multi drug efflux pump component CmeC. Two iron acquisition proteins ChuA and CfrB were also identified as well as the membrane associated flagellar component, FlgL, the flagellar hook protein (FlgE), hook-associated protein (FlgH) and both flagellin proteins, FlaA and FlaB, which are enriched under the same conditions as OM proteins [22].

Three lipoproteins, which are encoded by a locus of three genes, were also identified (CJJ81176_0124, CJJ81176_0125 and CJJ81176_0126). Oakland et al. [51] recently characterised homologues of these lipoproteins in strain NCTC11168 (Cj0089, Cj0090, and Cj0091). Few autotransporter proteins are annotated in *C. jejuni* yet they are common features of Gram-negative bacterial outer membranes. In this study we detected the expression of one putative autotransporter, the serine protease CJJ81176_1367. Bioinformatic analysis of this protein shows several features of autotransporters including the β-strand repeat (IPR013425) and β-barrel domain (IPR005546).

The roles of Omp18 (CJJ81176_0148), Omp85 (CJJ81176_0164) and the putative organic solvent tolerance protein (CJJ81176_1268) have not been investigated in *C. jejuni* although homologues of these proteins in other bacteria are involved in maintenance of membrane integrity and biogenesis. Omp18 is also known as peptidoglycan associated protein (Pal), which is a component of the well characterised Tol-Pal envelope complex [52]. Within *Campylobacter* genomes, Omp85 is the closest homologue of BamA, which in other Gram-negative bacteria is the OM component of the β-barrel assembly complex [25]. The protein annotated as a putative organic solvent tolerance protein is the closest *Campylobacter* homologue to *E. coli* LptD (*LP*S *t*ransport) [53]. Disruption of a *Helicobacter pylori* homologue, OstA (29% sequence identity with CJJ81176_1268) resulted in an increase in membrane permeability, susceptibility to hydrophobic and β-lactam antibiotics and sensitivity to organic solvents [54] and possibly performs a similar role in *C. jejuni*.

Eleven further gene translation products were also identified, including nine uncharacterised proteins of chromosomal origin and two plasmid encoded proteins – VirB9, and the hypothetical protein CJJ81176_pVir0048.

Several proteins identified in this study are by consensus considered to be cytoplasmic, although homologues of superoxide dismutase (SOD), thiol peroxidase (Tpx), bacterioferritin (Dps), gamma-glutamyltransferase (GGT) and l-asparaginase have been localised to the OM in the closely related *H. pylori* 55, 56, 57, 58. Esposito et al. [59] speculated that the localisation of SOD in *H. pylori* may be due to an extended C-terminal tail, a feature shared with *Campylobacter* SOD. Protein CJJ81176_1519 is annotated as a putative bacterioferritin although was characterised by Ishikawa et al. [60] as a Dps (DNA protection during starvation) protein, a protein class which is widely distributed in bacterial species and are members of the ferritin superfamily. The protein with highest sequence similarity to CJJ81176_1519 outside *Campylobacter* spp. is NapA of *H. pylori*, a surface exposed adhesin [61]. GGT, a component of the antioxidant glutathione pathway, is required for persistent colonisation of the avian intestine by *C. jejuni* 81116 [62] although GGT is not present in all *C. jejuni* isolates, including strains NCTC11168 and RM1221. Elongation factor Tu (EF-Tu) is central to protein synthesis in the cytoplasm although it is now considered to play a variety of roles within the cell and has been localised to the OM of Gram-negative bacteria, including *Neisseria meningitidis* [63] as well as the surface of *Mycoplasma pneumoniae* and *Lactobacillus johnsonii* where it is thought to contribute to host adhesion 64, 65, 66. Thus, as increasingly shown for other bacteria, *C. jejuni* may express non-classically associated OM “moonlighting” proteins on its surface with potential roles in infection.

### 3.2 Suggested re-annotation of *C. jejuni* strain 81-176 open reading frames

The annotation of CJJ81176_1108 as a putative lipoprotein and presence of β-barrel domain of CJJ81176_1268 suggest OM association of both proteins although their annotated amino acid sequences lack signal peptides. Closer examination of the genome sequences upstream from the annotated sequences within the 81-176 genome (NC_008787) for CJJ81176_1108 and CJJ81176_1268 revealed alternative translational start sites for these sequences, resulting in an additional N-terminal 56 and 7 amino acids respectively. These alternative N-terminal sequences contain intact lipoprotein signal peptides and are annotated as translational start sites for orthologues in other *C. jejuni* subsp. *jejuni* genomes in NCBI databases. Furthermore MS data searched against the revised sequence for CJJ81176_1108 identified a peptide indicating expression of this region of the protein. This evidence suggests that the translational start codons for accession entries gi|121612654 and gi|121504146 are currently mis-annotated; the suggested revised N-terminal sequences are shown Figure S1 of the supplementary data.

### 3.3 Distribution of proteins across *Campylobacter* species

**Fig. 1:**
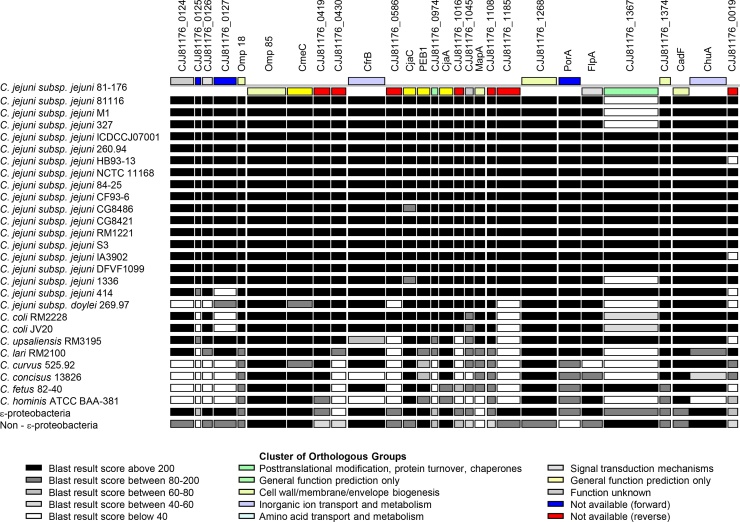
Comparison of *C. jejuni* 81-176 OM protein amino acid sequences identified by LC–ESI-MS/MS with amino acid sequences derived from 27 selected *Campylobacter* genomes and specified NCBI genomic datasets, using the BLASTP algorithm. Reciprocal best hits are identified and represented graphically with the strength of homology shown as shaded rectangles. Colour coding of *C. jejuni* 81-176 query sequences corresponds to Cluster of Orthologous Groups (COGs), allocated by National Center for Biotechnology Information (NCBI) which indicates predicted function.

We used in-house software to examine conservation and sequence similarity of a selected panel of characterised and uncharacterised OM proteins within *Campylobacter* genomes. A graphical representation of sequence similarity is provided, simultaneously facilitating the identification of conserved determinants that may be important in *Campylobacter* fitness and pathogenicity, heterogenous coding sequences, and highlights the presence of potential species/strain-specific markers. Fig. 1 shows comparisons for selected OM proteins against *Campylobacter* species and strains and selected NCBI datasets. This analysis demonstrates that the majority of OM proteins are conserved amongst *C. jejuni* subsp. *jejuni* strains, with the exception of the serine protease CJJ81176_1367 and hypothetical protein CJJ81176_0019 (discussed in more detail in the next section). The results also indicate that there is little sequence similarity between *C. jejuni* OM proteins and proteins within *Campylobacter* spp. other than *C. coli*. The majority of OM proteins shared high sequence similarity with proteins present within both strains of *C. coli* although the lipoprotein CJJ81176_0125, CJJ81176_1185 (Omp50), the hypothetical protein CJJ81176_0127 and the serine protease CJJ81176_1367 are absent. Several OM proteins of strain 81-176 were revealed to have high sequence similarity (up to 91%) with *C. upsaliensis* and/or *C. lari* proteins, although many OM proteins were divergent or absent. Only a few protein sequences are conserved in *Campylobacter* spp. *curvus*, *concisus*, *fetus* and *hominis* and the remaining proteins are either divergent or absent completely. Results of BLASTP searches of selected NCBI databases reveals presence of conserved proteins within *Epsilonproteobacteria* class (excluding the *Campylobacter* genus), as well as more widely amongst bacteria.

### 3.4 Analysis of sequence variability of OM proteins within *C. jejuni* isolates

**Fig. 2:**
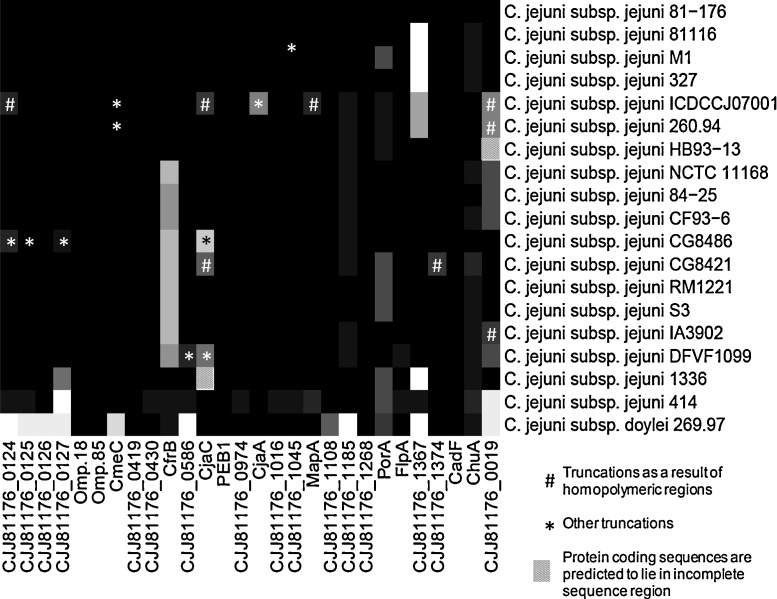
Smith–Waterman analysis of sequence similarity. Image represents scores for Smith–Waterman global alignments, which were carried out between protein pairs. Scores take account of the disparities in protein lengths and are displayed as a heat map (black = 100% and white = 0% identity). Regions of incomplete sequence and protein truncations are indicated.

Given the reported genetic variation within *Campylobacter* strains and species [24], conservation and sequence similarity of OM proteins in *C. jejuni* genomes were further investigated. The Smith–Waterman algorithm was used to generate alignments, instead of the time-optimised BLAST algorithm, and normalised scores were displayed as a heat map to allow greater discrimination (Fig. 2). Amino acid sequences for CadF, CJJ81176_1268, Omp18/Pal, CJJ81176_0126, and the uncharacterised proteins CJJ81176_0430, CJJ81176_0974, CJJ81176_1016, CJJ81176_1108, and CJJ81176_0419 are all highly conserved across *C. jejuni* subsp. *jejuni* isolates, which suggests that these are essential membrane proteins. Omp85, PEB1, and FlpA were also found to be conserved, although some sequence variations exist, all of which are predicted to be a result of assignment of alternative start codons. Alternatively assigned start codons are shown in Table S7 of the supplementary data.

Several proteins showed divergence across strains with respect to sequence heterogeneity as well as presence/absence. PorA, ChuA, and CJJ81176_1185 (Omp50) homologues exist in all *C. jejuni* subsp. *jejuni* strains but the Smith–Waterman scores indicate divergence in sequence; closer inspection of sequence alignments shows all three proteins contain regions of variability. This is of particular interest for Omp50, which was recently shown to coordinate capsule formation *via* a tyrosine phosphorylation cascade [17]. Whether this sequence variability influences protein function warrants further study. The variability of PorA is well documented and has been exploited in the development molecular epidemiology typing methods 67, 68, 69, 70. The serine protease autotransporter CJJ81176_1367 is absent in strains M1, 81116, and 327. Additionally, a divergent sequence is present within strains ICDCCJ07001 and 260.94, although this sequence shares stronger sequence similarity with CJJ81176_1376, an additional sequence within the *C. jejuni* 81-176 genome. CJJ81176_0019 is divergent in strains NCTC11168, DFVF1099, CF93-6 and 84-25 and strains ICDCCJ07001 and 260.94 contain truncated sequences (see below). The sequence is also absent from the genome of strain HB93-13 although the region is towards a contig boundary, hence truncation may be due to incomplete sequence information.

Amino acid sequences of CmeC, CjaC, CjaA, MapA, the lipoproteins CJJ81176_0124, CJJ81176_0125 and CJJ81176_1045 and the uncharacterised proteins CJJ81176_0019, CJJ81176_0127 and CJJ81176_0586, are conserved in the majority of *C. jejuni* subsp. *jejuni* strains although in some strains either truncations or extended sequences are present due to frameshifts or substitutions which result in stop codons. A summary of truncations is given in Table S8 of the supplementary data and indels are indicated. Such frameshifts may be of biological importance as a truncated version of the protein may result in altered virulence, metabolic capability or survivability. Some truncations are of considerable size, and in some cases the resulting protein sequences are annotated as pseudogenes although annotation of some ORFs in different strains is inconsistent. Some proteins are found to be truncated in many strains; truncations of CjaC are found within five strains, all of which appear to be due to frameshifts. Diversity within CjaC has been reported previously [71]. Many truncations were observed to occur as a result of insertions/deletions within homopolymeric tracts. Notably, the tracts are A or T rather than G or C which is more commonly documented in *Campylobacter* spp. Although it is tempting to speculate that these A or T homopolymeric tracts may allow a rapidly adaptive and reversible alteration in OM protein composition and a potential survival/adaptation strategy, it is more likely that many of these indels are a result of the high error rate in high throughput sequencing of homopolymeric regions.

Iron availability is a critical requirement for bacteria, resulting in diverse uptake mechanisms. Several iron acquisition systems have been identified in *C. jejuni*, many with OM components. Variation in distribution of these systems is well documented and is discussed in a review by Miller et al. [18]. Sequence analysis of the ferric enterobactin receptor CfrB also reveals a degree of variation within coding sequences for this protein. An intact coding sequence for CfrB is present within strains ICDCCJ07001, 260.94, HB93-13, 81116, M1, and 327. Frameshifts resulting in truncated proteins in strains 84-25, DFVF1099 and CF93-6 were also observed. Manual inspection revealed that within NCBI protein sequence databases for strains NCTC11168, RM1221, S3, IA3902, CG8421 and CG8486, the protein with closest sequence similarity to CfrB is the iron binding protein CfrA, which is not found within the strain 81-176 genome. Despite the absence of annotated protein sequences within the databases held at NCBI for several strains, homologous nucleotide sequences similar to *cfrB* are present in all genomes, although several sequences contain indels which results in their exclusion from annotated protein sequence databases. A summary of frameshifts and resulting outcomes is shown in Table S9 of the supplementary data. Significantly, all genomes with truncated CfrB proteins contain an intact coding sequence for CfrA and conversely, genomes with highly similar CfrB sequences lack CfrA indicating a greater conservation in strains without the alternative CfrA system.

Divergence in environmental isolates and *C. jejuni* subsp. *doylei* 269.97 is also apparent. Strain 414 and, to a lesser extent, strain 1336 show divergence in several proteins which are conserved in other *C. jejuni* subsp. *jejuni* strains. Fig. 2 shows that several proteins conserved within *C. jejuni* subsp. *jejuni* isolates are absent from *C. jejuni* subsp. *doylei*. *C. jejuni* subsp. *doylei* is associated with both gastritis and enteritis and more commonly bacteraemia, particularly in paediatric patients, and has been frequently isolated from blood cultures [72].

## 4 Concluding remarks

In 2008, Cordwell et al. carried out a comprehensive investigation of the membrane compartment of *C. jejuni* although this analysis was not restricted to the OM as it included the inner membrane and periplasmic space [30]. Membrane-associated proteins are typically hydrophobic, thus in order to investigate the proteome of the OM compartment of *C. jejuni* 81-176, we enriched Sarkosyl-insoluble bacterial proteins and applied a gel-based proteomics approach. Characterisation of the OM compartment is paramount to understanding pathogenicity, and it is also important to establish the distribution of these factors within strains. Equally, proteins conserved within disease-causing species which are absent in non-pathogenic species suggest a potential requisite for pathogenicity. We also investigated conservation of a panel of OM proteins within selected genomic datasets. Whilst it is apparent that individual isolates may contain a diversity of proteins within the OM, it is also clear that a number of proteins are conserved; a large proportion of these remain functionally uncharacterised, however.

The similarities between *C. jejuni* and *H. pylori* OM emphasise a requirement for further characterisation of *Epsilonproteobacteria* OM, for which common biological processes are already known to differ from those of the well-studied Enterobacteriaceae [73]. Since protein content varies both quantitatively and qualitatively in response to environmental cues, future efforts will examine homogeneity and heterogeneity of protein expression within and between strains under different culture conditions and phases of growth, in response to specific stimuli and upon interaction with hosts. The results presented here reinforce the notion that *C. jejuni* should be considered as heterogeneous bacteria with potential for considerable variability at the genotypic, phenotypic and ultimately pathogenic levels.

## Conflict of interest

All the authors have reported that they have received grants from Scottish Government/RESAS and BBSRC during the conduct of the study.

## Transparency document

Transparency document

## Figures and Tables

**Table 1:** Genomes used in this study.

Bacteria	Source	Accession		Ref.	Genome status
		Genbank	RefSeq		
**Query sequences**					
*C. jejuni* subsp. *jejuni* 81-176	Contaminated milk	CP000538	NC_008787	[37]	Complete
			NZ_AASL00000000	[74]	Draft
*C. jejuni* subsp. *jejuni* 81-176 pVir		CP000550	NC_008770		Complete
*C. jejuni* subsp. *jejuni* 81-176 pTet		CP000549	NC_008790	–	Complete

**Subject sequences**					
*C. jejuni* subsp. *jejuni* 1336	Water/wildlife isolate	CM000854	NZ_ADGL00000000	[75]	Draft
*C. jejuni* subsp. *jejuni* 260.94	GBS strain from the Red Cross Children's Hospital in Cape Town, South Africa	AANK01000000	NZ_AANK00000000	–	Draft
*C. jejuni* subsp. *jejuni* 327	Turkey skin surface	ADHM00000000	–	[76]	Draft
*C. jejuni* subsp. *jejuni* 414	Bank vole	ADGM00000000	NZ_ADGM00000000	[75]	Draft
*C. jejuni* subsp. *jejuni* 81116	Waterborne outbreak	CP000814	NC_009839	77, 78	Complete
*C. jejuni* subsp. *jejuni* 84-25	Cerebrospinal fluid of a child with meningitis can go systemic	AANT00000000	NZ_AANT00000000	–	Draft
*C. jejuni* subsp. *jejuni* CF93-6	MFS patient in Japan	AANJ00000000	NZ_AANJ00000000		Draft
*C. jejuni* subsp. *jejuni* CG8421	Bloody diarrhoea patient in Thailand	ABGQ00000000	NZ_ABGQ00000000	[79]	Draft
*C. jejuni* subsp. *jejuni* CG8486	Inflammatory diarrhoea patient in Thailand	AASY00000000	NZ_AASY00000000	[80]	Draft
*C. jejuni* subsp. *jejuni* DFVF1099	Isolated from chicken slaughterhouse	ADHK00000000	–	[81]	Draft
*C. jejuni* subsp. *jejuni* HB93-13	Faeces of 8-year-old boy in China with acute motor axonal neuropathy form of GBS	AANQ00000000	NZ_AANQ00000000	–	Draft
*C. jejuni* subsp. *jejuni* IA3902	Sheep abortion	CP001876	–	[82]	Complete
*C. jejuni* subsp. *jejuni* ICDCCJ07001	GBS patient	CP002029	NC_014802	[83]	Complete
*C. jejuni* subsp. *jejuni* M1	Diarrheic patient	CP001900	–	[84]	Complete
*C. jejuni* subsp. *jejuni* NCTC11168	Diarrheic patient	AL111168	NC_002163	[24]	Complete
*C. jejuni* subsp. *jejuni* RM1221	Skin of a retail chicken	CP000025	NC_003912	[85]	Complete
*C. jejuni* subsp. *jejuni* S3	Chicken faeces	CP001960	–	[86]	Complete
*C. jejuni* subsp. *doylei* 269.97	Blood of bacteremia Patient	CP000768	NC_009707	–	Complete
*C. coli* RM2228	Chicken carcass	AAFL01000000	NZ_AAFL00000000	[85]	Draft
*C. coli* JV20	Multi-drug-resistant chicken isolate	AEER01000000	NZ_AEER00000000	[85]	Draft
*C. upsaliensis* RM3195	GBS patient	AAFJ01000000	NZ_AAFJ00000000	[85]	Draft
*C. lari* RM2100	Clinical isolate	CP000932	NC_012039	[85]	Complete
*C. curvus* 525.92	Gastrointestinal clinical isolate	CP000767	NC_009715	–	Complete
*C. concisus* 13826	Gastrointestinal clinical isolate	CP000792	NC_009802	–	Complete
*C. fetus* 82-40	Blood of a renal transplant patient	CP000487	NC_008599	–	Complete
*C. hominis* ATCC BAA-381	Faeces of healthy human	CP000776	NC_009714	[87]	Complete

**Table 2:** Proteins identified by LC–ESI-MS/MS analysis of *C. jejuni* 81-176 OM. Number of non-redundant peptides and sequence coverage are given for each biological replicate.

Locus tag	NCBI annotation	Gene	No. of peptides			% Coverage			Mr
			1	2	3	1	2	3	
CJJ81176_0019	Conserved hypothetical protein	–	6	3	1	36.4	19.2	5.1	24.3
CJJ81176_0025	Flagellar hook protein FlgE	*flgE*	35	35	32	65.9	65.4	58.6	89.4
CJJ81176_0056	l-Asparaginase	*ansA*	9	9	8	41.4	31	36.5	36.9
CJJ81176_0067	Gamma-glutamyltransferase	*ggt*	8	5	8	18.5	10.1	18.5	60.3
CJJ81176_0113	l-Lactate permease	*lctP*	3	1	4	8.4	3.6	9.5	60.1
CJJ81176_0116	Cytolethal distending toxin, subunit A	*cdtA*	3	–	3	12.3		12.3	29.9
CJJ81176_0124	Lipoprotein, putative	–	4	3	7	12.8	8.2	20.5	51.3
CJJ81176_0125	Lipoprotein, putative	–	3	3	4	28.7	27	38.5	14.1
CJJ81176_0126	Lipoprotein, putative	–	10	10	10	53.1	53.1	53.1	22.4
CJJ81176_0127	Hypothetical protein	–	8	15	15	29.7	52.1	49.7	49.3
CJJ81176_0148	Peptidoglycan-associated lipoprotein Omp18	–	5	5	5	33.9	33.9	33.9	17.8
CJJ81176_0164	Outer membrane protein, OMP85 family	–	15	13	18	24.8	23.8	31.9	83.1
CJJ81176_0205	Superoxide dismutase, Fe	*sodB*	3	1	4	14.1	7.7	21.8	24.8
CJJ81176_0356	Antioxidant, AhpC/Tsa family	*ahpC*	4	2	1	29.8	17.7	11.6	21.9
CJJ81176_0388	RND efflux system, outer membrane lipoprotein CmeC	*cmeC*	7	7	4	22.6	21.3	15	55.4
CJJ81176_0419	Lipoprotein, putative	–	4	6	4	18.7	32.2	19.3	37.3
CJJ81176_0430	Lipoprotein, putative	–	3	1	8	11.4	3.7	32.8	33.2
CJJ81176_0471	TonB-dependent receptor, putative, degenerate	*cfrB*	5	10	10	10.1	21.4	21.3	79.2
CJJ81176_0499	Translation elongation factor Tu	*tuf*	4	1	3	19.5	5	12.8	43.6
CJJ81176_0586	Conserved hypothetical protein	–	5	7	7	20.1	30.7	30.4	35.0
CJJ81176_0641	Nonheme iron-containing ferritin	*ftn*	9	8	8	62.3	58.7	58.7	19.5
CJJ81176_0710	Flagellar L-ring protein FlgH	*flgH*	2	4	4	15.1	27.2	34.1	25.2
CJJ81176_0757	cjaC protein	*cjaC*	8	7	10	47.4	36.3	45	27.8
CJJ81176_0800	Thiol peroxidase	*Tpx*	3	4	5	24	34.9	46.3	18.4
CJJ81176_0894	Flagellin family protein	*flgL*	5	7	7	10.7	14.8	14.1	81.9
CJJ81176_0928	Amino acid ABC transporter, periplasmic amino acid-binding protein PEB1	*pebA*	5	–	4	30.5	–	23.6	28.1
CJJ81176_0974	Conserved hypothetical protein	–	6	6	6	53.5	54.2	43.1	16.2
CJJ81176_1001	CjaA protein	*cjaA*	8	7	10	36.2	31.9	46.2	30.9
CJJ81176_1016	Conserved hypothetical protein	–	2	4	2	13.2	23.7	13.2	20.5
CJJ81176_1045	Lipoprotein, putative	–	5	7	6	52	41.5	55.6	18.5
CJJ81176_1048	Outer membrane lipoprotein MapA	*mapA*	7	8	7	40.2	51.9	42.1	24.1
CJJ81176_1108^a^	Lipoprotein, putative	–	2	2	2	26.3	26.3	26.3	12.7
CJJ81176_1185	Conserved hypothetical protein		4	9	9	15.7	27.7	23.6	52.6
CJJ81176_1204	Methyl-accepting chemotaxis protein	–	4	3	8	30.9	23	52.7	19.3
CJJ81176_1268^a^	Organic solvent tolerance protein, putative	–	–	2	3	–	4.6	6.5	79.6
CJJ81176_1275	Major outer membrane protein	*porA*	19	21	18	66.3	65.8	64.6	45.7
CJJ81176_1295	Fibronectin type III domain protein	*flpA*	14	13	11	47.3	45.6	41.7	46.1
CJJ81176_1338	Flagellin	*flaB*	26	25	24	65.8	64.4	58.7	59.7
CJJ81176_1339	Flagellin	*flaA*	29	28	27	66.5	68.1	62.3	59.5
CJJ81176_1367	Serine protease, subtilase family	–	3	1	5	5.5	1.2	5.8	116.2
CJJ81176_1374	Lipoprotein, VacJ family		2	1	2	9.5	4.7	9.5	26.4
CJJ81176_1471	Fibronectin-binding protein	*cadF*	11	11	11	48	43.6	42.3	36.0
CJJ81176_1519	Bacterioferritin, putative	–	5	4	4	47.7	35.6	38.3	17.2
CJJ81176_1601	TonB-dependent heme receptor	*chuA*	7	8	16	13.7	15.8	33	80.0
CJJ81176_1690	Ribosomal protein S8	*rpsH*	3	–	4	35.9	–	42	14.7
CJJ81176_pVir0002	VirB9	–	1	2	3	3.7	7.6	11.2	40.8
CJJ81176_pVir0048	Conserved hypothetical protein	–	4	2	5	39.2	21.5	47.7	15.0

a Sequences with re-assigned start codons were used.
