# Supplementary material for: Proteomic and genomic analysis reveals novel Campylobacter jejuni outer membrane proteins and potential heterogeneity
Source: EuPA Open Proteom. 2014 Jun 26;4:184–94. doi: 10.1016/j.euprot.2014.06.003 (PMC4975774; doi:10.1016/j.euprot.2014.06.003)
Supplement: Supplementary file 2 [file mmc1.docx]

atgtggcgtaaattttccctattgctaggaactagcatagctttaaatgcagcacaagttgat

1 M W R K F S L L L G T S I A L N A A Q V D 17

atgaaaaagattttaattttttgtattggtttatttttaggtgcttgtggatatattcctaca

M K K I L I F C I G L F L G A C G Y I P T

tcaaaaatcgctaataatattttcgatgagaaagtttatgttaatgttgaattaagtcaacaa

S K I A N N I F D E K V Y V N V E L S Q Q

gacccaaaaaatagtatctatgttgccgatactttaaaagaaatggtaatttcaaaattaggg

D P K N S I Y V A D T L K E M V I S K L G

cgtaagcttgctttaaaacatgaggcagatgatgtaattaatgttaaaatgaataatttagaa

R K L A L K H E A D D V I N V K M N N L E

tttattcctttagcttatgataaaaatggttatgtcattagttataaagctaaattaaattta

F I P L A Y D K N G Y V I S Y K A K L N L

gattttaatgttgtctttaaagatggttcaagtcaggcttttagtacaagtggaagttataat

D F N V V F K D G S S Q A F S T S G S Y N

tttgaaatttctccaaatagtattattagtgattctgcaaggtatgaggctattcgagcagct

F E I S P N S I I S D S A R Y E A I R A A

Tcaagcgaggcatttgatgagtttatttccgtaattgctattaaagggcaaaaacgtgattcc

S S E A F D E F I S V I A I K G Q K R D S

aaatactaa

K Y -

#

*

*

#

**A**

**B**

Figure S1.

Nucleotide and amino acid sequence for CJJ81176_1108 (A) and N-terminal sequence of CJJ81176_1268 (B) within genome NC008787 (YP001000768). Start codons assigned by NCBI (hash) and revised start codons (asterisk) are indicated. Signal peptidase II (A) and I (B) most likely cleavage site is indicated (arrow). Sequence coverage of CJJ81176_1108 by LC-ESI MS/MS is highlighted (grey) indicating detection of three peptides upstream from currently designated peptide.
